# Characterization of Active Anthocyanin Degradation in the Petals of *Rosa chinensis* and *Brunfelsia calycina* Reveals the Effect of Gallated Catechins on Pigment Maintenance

**DOI:** 10.3390/ijms18040699

**Published:** 2017-03-25

**Authors:** Honghui Luo, Shuangfan Deng, Wei Fu, Xin Zhang, Xuelian Zhang, Zhaoqi Zhang, Xuequn Pang

**Affiliations:** 1 State Key Laboratory for Conservation and Utilization of Subtropical Agro-Bioresources, South China Agricultural University, Guangzhou 510642, China; hhluo@stu.scau.edu.cn (H.L.); dengshuangfan@nibs.ac.cn (S.D.); weifu996429825@163.com (W.F.); zhang_ivy@163.com (X.Z.); xuelianzhang@scau.edu.cn (X.Z.); 2 College of Life Sciences, South China Agricultural University, Guangzhou 510642, China; 3 College of Horticulture, South China Agricultural University, Guangzhou 510642, China; 4 Guangdong Provincial Key Laboratory of Postharvest Science of Fruits and Vegetables, Guangzhou 510642, China

**Keywords:** anthocyanin degradation enzyme activities, tannins, anthocyanin maintenance, enzyme inhibition

## Abstract

Anthocyanin degradation decreases ornamental or nutritional values of horticultural products. To investigate factors that may influence colour change in flower development, anthocyanin degradation was compared between the flowers of *Brunfelsia calycina* and *Rosa chinensis*, which show rapid and slow degradation, respectively. In-gel activity assays, high performance liquid chromatography (HPLC) analysis of tannins, enzyme kinetics measurement and immune-detection of anthocyanin degradation related-perioxidases (PODs) were carried out for the comparison. Rose petals possessed significantly lower anthocyanin degradation-related POD activities than *Brunfelsia* petals, which may be related to the high tannin contents. Epicatechin gallate (ECG) and gallocatechin gallate (GCG) were detected in rose as 161.3 ± 12.34 and 273.56 ± 41.23 μg/g FW (Fresh Weight) respectively, while not detected in *Brunfelsia*. ECG and GCG inhibited the activities of the *Brunfelsia* POD with half maximal inhibitory concentrations (IC50s) as 21.5 and 29.7 μM respectively, and increased the colour intensities of the anthocyanins. Catechin and epicatechin did not inhibit the POD activity, while serving as POD substrates, with *K*m (the Michaelis constant) as 0.48 and 1.23 mM. Similar protein levels of the anthocyanin degradation-related 40-kDa PODs were detected in *Brunfelsia* and rose. In summary, high amount of tannins, particularly ECG and GCG, in red rose petals may inhibit the degradation-related enzymes, leading to the maintenance of anthocyanins in vivo.

## 1. Introduction

Anthocyanins are the most important flavonoid pigments widely distributed in nature, dressing the flowers of plants with colours ranging from orange, pink, red, magenta, purple, and blue to blue-black [1]. The attractive colours contributed by anthocyanins facilitate the reproduction of flowering plants, and have long been admired and used by humans to beautify their environment. Recently, the health benefits of the pigments as dietary components providing protection against a wide range of human diseases have drawn even more interest than the colourful appearance they provide to the plant tissues [2,3].These pigments accumulate in the vacuoles and their stability and hue depend on intravacuolar conditions such as pH, the concentration of the pigments, copigmentation with coexisting colourless flavonoids, and formation of complexes with metal ions. The maintenance of pigment concentration is determined by both the biosynthesis and degradation of anthocyanin in plants. So far, there is detailed molecular information available on anthocyanin synthesis [4], while there is little information on the stability and catabolism of anthocyanins in plants [5].

Anthocyanin degradation has been investigated mainly in fruits and a few ornamental plants [5,6,7,8]. Based on the studies of pigment loss in fruits, juice and wine [9,10,11], polyphenol oxidase (PPO) and perioxidase (POD) are felt to be the main enzymes involved in anthocyanin degradation in fruit extracts [10,12]. It was presumed that PPO/POD first oxidizes other phenolic compounds to form quinones, which are unstable molecules which then react with anthocyanins, resulting in decolouration, browning and decrease in anthocyanin content [7,13,14]. PPO and POD activities were detected in harvested bayberry (*Myrica rubra*) [15] and strawberry [16] fruits. Recently, we identified that an anthocyanin degradation enzyme/laccase (ADE/LAC) was responsible for the anthocyanin degradation during Litchi pericarp browning. The laccases degraded anthocyanins in a similar enzyme–phenol–anthocyanin model to PPOs and shared some substrate specificity range with PPOs, such as catechol and 2,2′-Azinobis-(3-ethylbenzthiazoline-6-sulphonate), ABTS. Protein sequencing and further enzymatic characterization are required to confirm whether the enzymes are laccases or PPOs [17].

Compared to the anthocyanin degradation studies in fruits, fewer investigations were carried out regarding the degradation mechanism in ornamental flowers. Active enzymatic anthocyanin degradation dependent on novel mRNA (messenger ribonucleic acid) and protein biosynthesis was found in *Brunfelsia calycina* (Hook.) Benth. flower petals, in which more than 90% of the anthocyanins degraded within 3 days after flower opening, leading to extremely rapidly colour changes from dark purple to completely white [8,18]. Recently, a basic class III peroxidase, BcPrx01, was found to be responsible for the in planta degradation of anthocyanins in *Brunfelsia calycina* [19]. BcPrx01 colocalizes with the pigments in the vacuoles of petals and has the ability to degrade complex anthocyanins. Both the mRNA and protein levels of BcPrx01 are greatly induced parallel to the degradation of anthocyanins.

In contrast to the rapid colour change and anthocyanin degradation in *B. calycina* flowers, relatively slower pigment degradation and colour change was observed for many other species. In miniature “KORcrisett” rose flowers, less than 25% of anthocyanins degraded from fully opened to senescent stage [20]. In pink petunia (*Petunia × hybrida* L. cv. Dreams Appleblossom), high values of anthocyanins were found at bud stage and decreased during flower opening, slightly increasing again at the last stage, during flower senescence [21]. The degradation of anthocyanins is dependent upon the pigment stabilities, which are influenced by tissue pH, environment temperature, presence of enzymes, light, oxygen, and copigmentation with other natural coexisting components and metal ion complexation in the vacuoles [22,23,24,25]. Comparison of plant tissues with different anthocyanin degradation patterns may help to understand the pigment degradation mechanism, or may help identify the factors in plants that influence the stabilities of anthocyanins.

In this study, to understand the factors that may influence anthocyanin degradation in flower petals, we chose two species whose flowers showed significantly different rates of colour change during flower development, *Brunfelsia calycina* and *Rosa chinensis* Jacq. In contrast to the rapid colour change in *B. calycina* flower petals, *R. chinensis* flowers show no obvious colour change in the same process. We compared ADE (anthocyanin degradation enzyme) activities and tannin contents in the petals of the two species. The negative correlation between the activities and the contents of tannins prompted us to further analyse the tannin constituent profiles in the petals and the putative effect of the catechin and its derivates on anthocyanin degradation-related POD activities. The immune-detection of PODs in the soluble and insoluble fractions of the enzyme extracts was also carried out to understand the different ADE activities in the tissues. The findings of this study may reveal that tannin substances that coexist with anthocyanins in plant cell vacuoles may protect the pigments from degradation and maintain the colour of flowers.

## 2. Results

### 2.1. Anthocyanin Degradation Was Found in B. calycina but Not in R. chinensis during Flower Development

**Figure 1:**
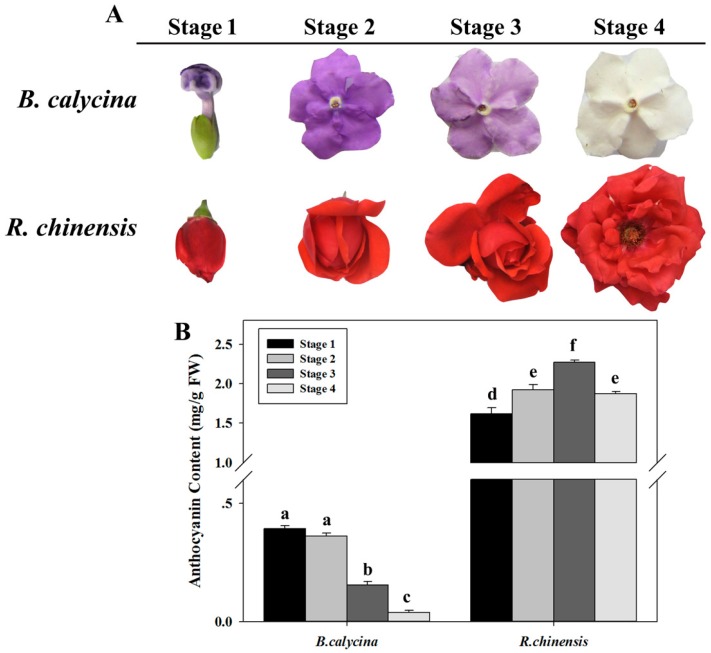
Change in the colour and anthocyanin contents during flower development of *Brunfelsia calycina* (Hook.) Benth. and *Rosa chinensis* Jacq. (**A**) The images of the flowers of two species. Stage 1 to 4 indicates flower buds, dark purple, light purple and white flowers respectively for *B. calycina*. For *R. chinensis*, the relevant stages represent flower buds, partially open, fully open and initially senescent flowers; (**B**) Change in anthocyanin content per gram fresh petals of *B. calycina* and *R. chinensis* flowers during the development. The values are means of the measurements of three individual extractions. Error bars indicate the standard error of mean (SEM) of the values. Different letters denote significant differences in the values according to unpaired *t* test (*p* < 0.05) while shared letters denote non-significant differences in the values.

The flower buds of *B. calycina* and *R. chinensis* opened with dark purple and dark red colours respectively, due to the accumulation of anthocyanins in the petals (Figure 1A). In the process of flower development, *B. calycina* petals rapidly lost their purple colour and turned completely white, whereas for *R. chinensis* flowers, the red colour was maintained. The petals were collected at stages 1 to 4, which represented flower buds, dark purple, light purple and white for *B. calycina*, and represented buds, partially opened, fully opened and initial senescent for *R. chinensis* (Figure 1A). For *B. calycina* flowers, anthocyanin contents decreased from 0.39 ± 0.01 to 0.04 ± 0.01 mg/g FW (Fresh Weight) during the process (Figure 1B). However, in the *R. chinensis* flowers, the anthocyanin concentration first increased from 1.62 ± 0.07 to 2.27 ± 0.03 mg/g FW and then slightly decreased to 1.87 ± 0.03 mg/g FW at stage 4 (Figure 1B). The results indicated rapid anthocyanin degradation occurred in *B. calycina* but not in *R. chinensis* during flower development.

### 2.2. High H_2_O_2_-Dependent Anthocyanin Degradation Enzyme Activity Was Found in the Petals of B. calycina but Not in R. chinensis during Flower Development

**Figure 2:**
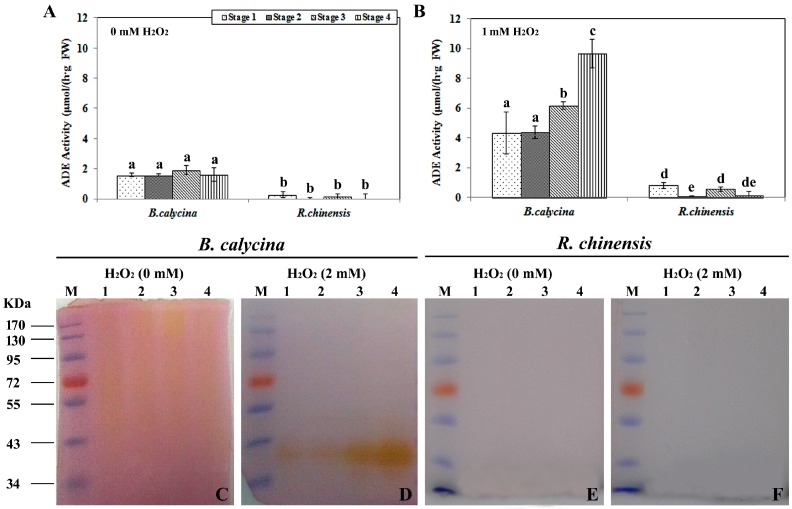
Anthocyanin degradation enzyme activities during flower development of *Brunfelsia calycina* and *Rosa chinensis*. (**A**) Anthocyanin degradation enzyme (ADE) activities in the petals of each species were detected with the anthocyanins purified from the individual flowers, at pH 4.0 without H_2_O_2_; (**B**) The ADE activities were detected as in A, but with 1 mM H_2_O_2_; (**C**–**F**) The ADE activities were also detected by in-gel activity assays with the same anthocyanin substrates as described above at pH 4.0, with or without H_2_O_2_. Lane M in each gel indicates the protein markers, while lanes 1 to 4 represent the samples from the stage 1 to 4, as indicated in Figure 1A,B. The statistical details of the values presented in (**A**,**B**) are as described in Figure 1.

To better understand the anthocyanin degradation mechanisms during flower development, ADE activities in the petals of *B. calycina* and *R. chinensis* were investigated. Anthocyanins were partially purified from the petals of the respective plants and used as the substrates for the ADE activity assay. Both H_2_O_2_-dependent and non-dependent ADE activities were monitored. Around 2 μmol/h/g FW of H_2_O_2_ non-dependent ADE activities were detected in *B. calycina* flowers at all the 4 stages, while less than 0.2 μmol/h/g FW was found for the activities in *R. chinensis* (Figure 2A). At least two-fold higher H_2_O_2_-dependent activities in *B. calycina* were detected compared to the H_2_O_2_ non-dependent ADE activities. The H_2_O_2_-dependent ADE activities increased at stage 3 and 4, whose activities were respectively 1.5- and 2.3-fold higher than the activities at stage 1 and 2 (Figure 2B).

The above ADE activity patterns obtained by the spectrophotometry method were further confirmed by the in-gel activity assays using the same anthocyanin substrates described above, with or without H_2_O_2_ (Figure 2C–F). The same amount of protein was loaded for each sample (Figure S1). Correlated to the above patterns (Figure 2A,B), weak H_2_O_2_ non-dependent ADE activity signals were observed for the *B. calycina* (Figure 2C). More intensive activity staining was observed in the presence of 2 mM H_2_O_2_ for *B. calycina*, particularly for the samples at the stages when the petals were turning white (Figure 2D). No activity staining was seen for any of the samples of *R. chinensis* flower with or without H_2_O_2_ (Figure 2E,F). Taken together, high H_2_O_2_-dependent ADE activity was detected to be positively correlated to anthocyanin degradation during *B. calycina* flower development, and minimal ADE activities in *R. chinensis* were correlated to minimal anthocyanin degradation during the same process of flower development.

### 2.3. Peroxidase and Polyphenol Oxidase Activities during the Flower Development

**Figure 3:**
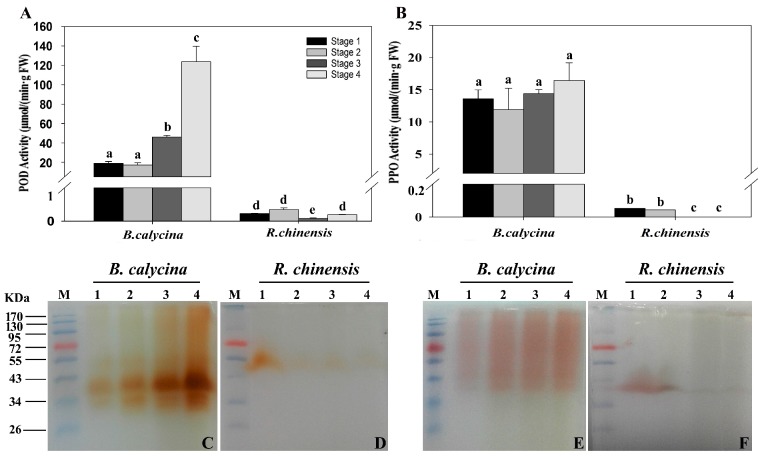
Peroxidase and polyphenol oxidase activities during flower development of *Brunfelsia calycina* and *Rosa chinensis*. (**A**) Activities of perioxidases (PODs) in the petals were measured with guaiacol and H_2_O_2_ as substrates at pH 7.0; (**B**) Activities of polyphenol oxidases (PPOs) were measured with 4-methylcatechol as substrate at pH 7.0; (**C**–**D**) Ten micrograms of total protein extract of different stages of each species was separated by semi-native polyacrylamide gel electrophoresis (PAGE) followed by guaiacol staining in the presence of 0.17 mM H_2_O_2_ for POD in-gel activity detection, while using 4-methylcatechol for PPO in-gel activity assay (**E**–**F**). The details of the values presented in (**A**,**B**) are as described in Figure 1, and the details of the lanes presented in (**C**–**F**) are as described in Figure 2.

The activities of POD and PPO in the petals were also determined using both spectrophotometry (Figure 3A,B) and the in-gel activity assay (Figure 3C–F). In *B. calycina* flowers, POD activities of close to 20 μmol/min/g FW were detected at stage 1 and 2, while 2.3- and 6.2-fold higher activities were detected respectively at pale purple and white stage, stages 3 and 4 (Figure 3A,C). Interestingly, the main POD activity staining, at close to 40 kDa (Figure 3C), was of the same intensity as the H_2_O_2_-dependent ADE activity staining (Figure 2D). Based on the requirement of H_2_O_2_ for POD activities, the H_2_O_2_-dependent ADE activity might be due to the activities of PODs. The PPO activities were detected using 4-methy catechol as the substrates, and were found to be almost the same intensity in all stages of *B. calycina* flowers, with slightly higher activities, around 16 μmol/min/g FW, at stage 4 (Figure 3B). Weak in-gel PPO activity bands were seen at stage 3 and 4, showing much less intensity than the POD activity bands (Figure 3E).

The POD and PPO activities detected in *R. chinensis* flowers were lower than those of *B. calycina* flowers. The *R. chinensis* POD activities decreased after stage 1, with the main activity staining at close to 50 kDa (Figure 3A,D). Low PPO activities were detected in at stage 1 and 2, and almost no activity was recorded at the later stages (Figure 3B,F). Together with the low ADE activity, the minimal anthocyanin degradation in *R. chinensis* flowers was correlated with the low anthocyanin degradation-related enzyme activities.

### 2.4. Low pH and High Levels of Tannins Were Found for the Anthocyanin Accumulating Cells of R. chinensis but Not B. calycina

**Figure 4:**
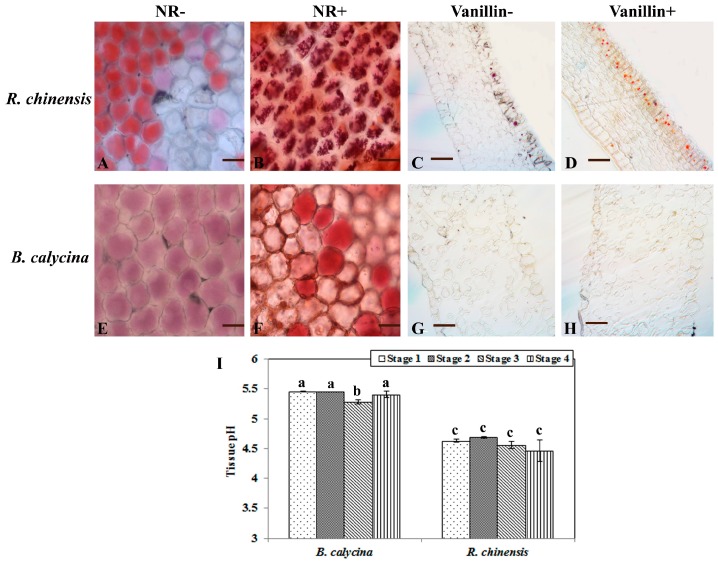
Tannin staining and pH of *Brunfelsia calycina* and *Rosa chinensis* flowers. (**A**,**B**,**E**,**F**) show the neutral red (NR) staining of the epidermal cells of *B. calycina* and *R. chinensis* petals. (**A**,**E**) are without staining (NR-), and (**B**,**F**) are with staining (NR+). Scale bars in (**A**,**B**,**E**,**F**) indicate 25 μm. (**C**,**D**,**G**,**H**) show the vanillin staining of the sections (2-mm thick) of the flower petals, (**C**,**G**) without vanillin treatment (Vanillin-), and (**D**,**H**) with vanillin treatment (Vanillin+). Scale bars in (**C**,**D**,**G**,**H**) indicate 100 μm. (**I**) The pH values of the homogenate of *B. calycina* and *R. chinensis* flower petals. The statistical details of the values presented in (**I**) are as described in Figure 1.

The colour and stability of anthocyanins are dependent on the pH and coexisting substances in the vacuoles, where anthocyanins accumulate. The petals were homogenized in water and the pH values were measured to predict the acidities of the petals. *B. calycina* petal homogenates were found to have a pH of 5.3 to 5.5, while a pH of 4.4 to 4.6 was determined for the *R. chinensis* flower samples (Figure 4I). The in vivo acidity of the anthocyanin accumulation cells in both plants was measured by staining the epidermal cells with neutral red (NR). The anthocyanin accumulation cells were observed in the epidermal cells of both plants before staining (Figure 4A,E). After staining with neutral red, *R. chinensis* cells exhibited much stronger accumulation of NR than the *B. calycina* cells, indicating that the *R. chinensis* cells were at lower pH (Figure 4B,F). In addition, orange staining by vanillin was also observed in the epidermal cells of *R. chinensis* petals, but not in the *B. calycina* cells (Figure 4D,H). The vanillin staining strongly indicates a high content of condensed tannins or proanthocyanidins accumulated in the *R. chinensis* but not in *B. calycina* epidermal cells.

### 2.5. Tannin Contents in the Flowers Was Found to be Related to the Preservation of Anthocyanins during Flower Development

**Figure 5:**
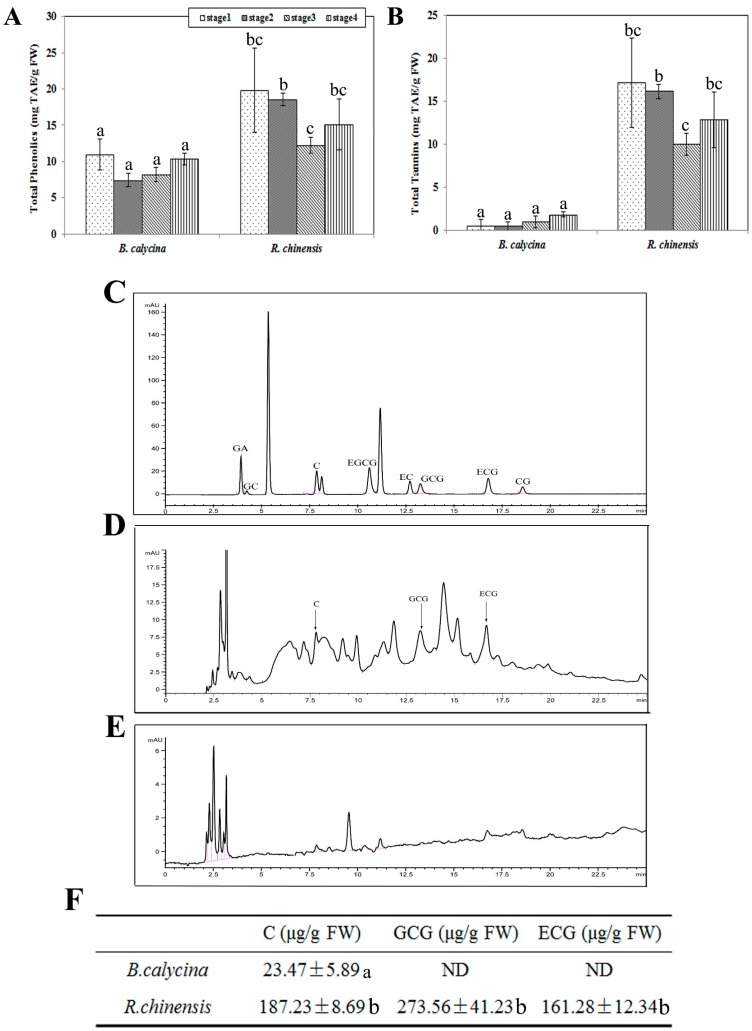
Contents of total phenolics and tannins and quantification of tannin constituents by HPLC. (**A**) Total phenolic contents as tannic acid equivalent (TAE) in the petals of *Brunfelsia calycina* and *Rosa chinensis* flowers; (**B**) Total tannin contents as TAE in the petals; (**C**) HPLC analysis of the tannin constituent standards; (**D**) HPLC analysis of the tannin extracts from *Rosa chinensis* petals at stage 2; (**E**) HPLC analysis of the tannin extracts from *Brunfelsia calycina* petals at stage 2; (**F**) The contents of the identified tannin constituents from HPLC. The statistical details of the values presented in (**A**,**B**,**F**) are as described in Figure 1. GA: gallic acid; GC: Gallocatechin; C: catechin; EGCG: epigallocatechin-3-gallate; EC: epicatechin; GCG: gallocatechin-3-gallate; ECG: epicatechin-3-gallate; CG: catechin gallate.

The different extent of vanillin staining of the anthocyanin accumulated cells prompted us to further analyse the total phenolic and tannin contents in the petals of the two flowers. Higher total phenolic contents were detected in the *R. chinensis* flowers than the *B. calycina* flowers at all stages (Figure 5A). The highest levels of total phenolics were found at stage 1 for both species, with 19.8 ± 5.78 and 11 ± 2.13 mg/g FW in *R. chinensis* and *B. calycina*, respectively. Interestingly, the tannins constituted more than 85% of the total phenolic compounds in *R. chinensis* flowers, while only 10% of the total phenolic compounds in the *B. calycina* flowers appeared to be tannins, with 12.8 ± 3.27 and 1.8 ± 0.3 mg/g FW respectively detected in *R. chinensis* and *B. calycina* petals at stage 4 (Figure 5B). Tannin contents were detected as ten-fold higher in the *R. chinensis* flowers than the *B. calycina* flowers at stage 3.

The content of five tannins components, namely, catechin and its derivates, epicatechin (EC), gallocatechin-3-gallate (GCG), epicatechin-3-gallate (ECG) and epigallocatechin-3-gallate (EGCG), were examined in the petals at stage 2 by high performance liquid chromatography (HPLC) (Figure 5C–F). Correlated to the low content of tannins in *B. calycina*, catechin itself was the only catechin derivate detected in those petals, at 23.47 ± 5.89 μg/g FW (Figure 5E,F). In *R. chinensis* petals, relatively high contents of ECG and GCG were detected as 161.28 ± 12.34 and 273.56 ± 41.23 μg/g FW, respectively (Figure 5C,D,F).

### 2.6. ECG and GCG, but not Catechin (C) and EC, Showed High Efficacy of Inhibition on POD Activities and Increased the Colour Intensity of the Anthocyanins In Vitro

**Figure 6:**
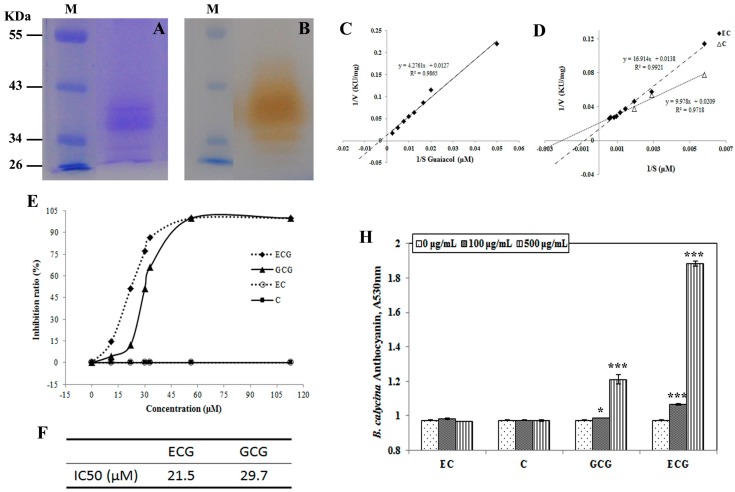

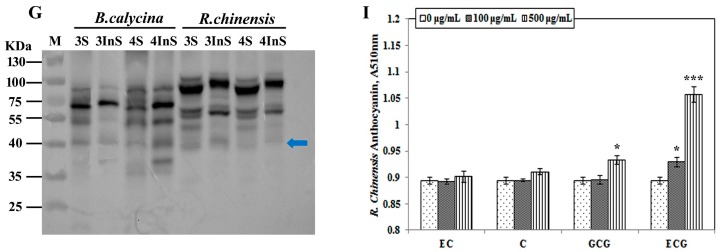
The effect of tannin constituents on the purified POD activity and the colour intensity of the anthocyanins. (**A**) sodium dodecyl sulfate—polyacrylamide gel electrophoresis (SDS-PAGE) analysis of the purified POD from *Brunfelsia calycina* after sequential diethylaminoethanol (DEAE)-Sepharose and Sephadex G-200 column chromatography, Lane M indicated the protein marker; (**B**) In-gel activity assay of the purified POD with guaiacol as substrate, Lane M was as in (**A**); (**C**) Lineweaver–Burk plot for the *K*m (the Michaelis constant) value detection of the purified POD to guaiacol; (**D**) Lineweaver–Burk plots for the *K*m values of the purified POD to epicatechin and catechin; (**E**) The inhibition effect of different tannin constituents on the POD activity; (**F**) The half maximal inhibitory concentration (IC50) values of ECG and GCG for the purified POD, which were estimated based on (**E**); (**G**) The immuno-detection of POD in both soluble and insoluble fraction proteins from *B. calycina* and *R. chinensis* at stages 3, 4. The blue arrow indicates the size of the POD band as 40 KDa; (**H**) The effect of different concentration of tannin constituents on the colour of anthocyanins from *B. calycina* flowers; (**I**) The effect of different concentration of tannin constituents on the colour of anthocyanins from *R. chinensis* flowers. An asterisk (* *p* < 0.05) represents the significance of difference between the addition of 0 and 100 or 500 μg/mL of tannin constituents. Three asterisks represent *p* < 0.001.

The tannin constituents in the petals that were identified by HPLC, namely C, EC, ECG and GCG were chosen to investigate their effect on the anthocyanin degradation in flowers. Due to the low anthocyanin degradation activities in *R. chinensis* petals, the anthocyanin degradation-related POD was purified from *B. calycina* by sequential diethylaminoethanol (DEAE)-sepharose and Sephadex G200 column chromatography (Figure S2). The purified POD fraction contained a major band of close to 40 kDa and the band showed activity with guaiacol (Figure 6A,B) in the presence of H_2_O_2_, indicating the POD was highly purified. The *K*m (the Michaelis constant) of the enzyme to guaiacol was detected as 336.7 μM at 25 °C (Figure 6C).

By using 400 μM guaiacol and 5 mM H_2_O_2_ as substrate, the POD activities were inhibited by the addition of ECG and GCG (Figure 6E). The half maximal inhibitory concentration (IC50) values of ECG and GCG for the POD were estimated to be 21.5 and 29.7 μM respectively (Figure 6F). No inhibition was detected for C and EC on POD activities (Figure 6E). Instead, C and EC were detected to be substrates for the POD, with the *K*m values detected as 477.42 and 1225.65 μM respectively (Figure 6D).

To understand the inhibition of ECG and GCG on the anthocyanin degradation-related POD activities in *R. chinensis* petals, POD proteins in both the soluble and insoluble fractions of the protein extracts of the petals were immune-detected by a polyclonal antibody raised against horseradish POD, which was predicted to be located in vacuole and of around 40 kDa. Protein bands of 40 kDa were detected at similar levels in both *R. chinensis* and *B. calycina* (Figure 6G). The results indicate that even though the 40-kDa POD protein seems to be present in the petals of *R. chinensis*, it may be inactivated by ECG and GCG.

Furthermore, increased intensity of the red colour of the anthocyanins was observed when more than 100 μg/mL of ECG or GCG was added to the anthocyanin solutions at pH 3.0 (Figure 6H,I). When 500 μg/mL GCG and ECG were respectively added into 0.05 mM anthocyanin solutions, 24.2% and 93.4% higher absorbance at 530 nm was respectively detected for the anthocyanins of *B. calycina*, and 4.5% and 18.3% higher absorbance at 510 nm was respectively detected for the anthocyanins of *R. chinesis*.

## 3. Discussion

Colour maintenance is important for the preservation of the market values of most ornamental flowers. Colour fading due to anthocyanin degradation decreases the ornamental value of the flowers of many species. The flowers of *B. calycina*, however, may be one of the exceptions in this case, whereby rapid degradation of anthocyanins leads to the coexistence of purple and white flowers in the same plant, adding to the ornamental value of the plants. Comparison of flowers showing rapid versus slow anthocyanin degradation may reveal the mechanisms of pigment degradation or maintenance, which is currently not as clear as the mechanism for the pigment biosynthesis. In the present study, we compared anthocyanin degradation of *Brunfelsia calycina* and *Rosa chinensis*, whose flowers respectively show dramatic and minimal colour change, during development. We found that, compared with *B. calycina*, *R. chinensis* showed extremely low anthocyanin degradation enzyme activities, which was closely related to the high tannin content in the petals.

Anthocyanin degradation is determined by the activities of degradation related enzymes and the stability of the pigments. Multiple enzymes were found to be involved in the in vivo anthocyanin degradation in plants [5]. We previously detected high H_2_O_2_-independent ADE activities in Litchi pericarp, which was responsible for the anthocyanin degradation during pericarp browning after the fruit harvest [7]. We purified and demonstrated that H_2_O_2_-independent ADE activities in Litchi pericarp was due to a laccase (LcADE/LAC) [17]. The anthocyanin degradation function of LcADE/LAC was further confirmed by its co-location with the pigment in vacuoles [17], In *Brunfelsia calycina*, an H_2_O_2_-dependent ADE activity was detected, and the enzyme was purified and demonstrated to be a peroxidase, BcPrx01, responsible for the in-plant degradation of anthocyanins, and BcPrx01 was also predicted to locate in vacuoles [19]. It was suggested that polyphenol oxidases were involved in the anthocyanin degradations in fruit juice [26]. Due to localization of PPOs in plasma and not co-localizing with anthocyanins, their function in anthocyanin degradation in vivo requires further study. In addition, although PPOs have long been considered to be involved in anthocyanin degradation, their activity must be differentiated from the activities that were attributed to laccases, since there is substrate specificity overlapping between laccases and PPOs. In the present study, both H_2_O_2_-dependent and -independent ADE activities were determined. For *B. calycina*, similar to the results reported by Zipor et al. [19], H_2_O_2_-dependent ADE activities due to the activities of BcPrx01 were detected and correspondingly increased with the colour loss of the petals. The H_2_O_2_-independent ADE activities, which may be due to the enzyme interaction with 4-methycatechol, was minor. Compared to the activities of *B. calycina*, extremely low activities of H_2_O_2_-dependent or -independent ADE, POD or PPOs were detected in the petals of *R. chinensis*, closely correlated to the minimal anthocyanin degradation in the flower petals.

Peroxidases are universal enzymes in various plant tissues [27]. Even though extremely low POD activities were detected in the petal of *R. chinensis*, a 40-kD POD protein isolate, which was predicted to be involved with anthocyanin degradation activities in *B. calycina* petals and to be located in the vacuole [19], was recognized by a POD antibody (Figure 6G), indicating the existence of pigment degradation-related POD proteins. Accordingly, effectors to PODs may exist in *R. chinensis* petals to inhibit the POD activities. Much higher levels of tannins were detected in *R. chinensis* than in *B. calycina* (Figure 5B). High levels of two gallated catechins, ECG and GCG were found in *R. chinensis* but not detected in *B. calycina*. Profile of metabolites in *B. calycina* petals during colour change was analysed by Bar-Akiva et al., and gallated catechins were not detected [18]. We found that ECG and GCG showed high efficacy of inhibition to the purified *B. calycina* POD with the IC50 values of 21.5 and 29.7 μM respectively. The biologically active constituents of tannins, including catechin and its derivates, EC, epigallocatechin (EGC), ECG and EGCG, are known to evoke a wide range of physiological responses [28,29]. Some catechins were reported to bind and inhibit some vital cellular enzymes, leading to suppression of certain biological processes [30,31,32,33,34]. EGCG inhibited the activity of phosphatase-1 (PP1) with IC50 values of 0.26–0.4 μM, via the interaction of EGCG with the catalytic subunit (PP1c) of PP1 [35]. Tea extracts containing CG, GCG, ECG and EGCG were good inhibitors of maltase, with IC50 values of 62, 67, 40 and 16 μM respectively, and were the potent inhibitors of α-glucosidase activity, while nongallated catechins were ineffective [36,37]. Fluorimetrically-determined binding constants for EGCG and ECG with catalase were observed to be 2.27 and 1.66 μM, respectively and exhibited good inhibition of pure catalase [38]. In the present study, ECG and GCG showed high inhibition efficacy on the *B. calycina* POD. Similar to the inhibition efficacy on the above described maltase [36], α-glucosidase [37] and catalase [38], the nongallated catechins, C and EC showed no inhibition of the *B. calycina* POD, and even appeared to be substrates for the enzyme. The catechins are biosynthesized by the reduction of leoanthocyanindins or anthocyanindins by leucoanthocyanidin reductase (LAR) or anthocyanindin reductase (ANR). High content of catechins were also found in anthocyanin-rich tissues [39,40]. Due to the co-localization of catechins and anthocyanins in vacuoles, the effect of these compounds on the maintenance of the pigments should be taken into account. Furthermore, higher intensity of the pigment was observed in vitro when ECG and GCG were added to the pigment solutions, particularly for the *B. calycina* anthocyanin solution, in which the natural content of these two catechins was negligible (Figure 5E,F). Taken together, high levels of gallated catechins in *R. chinensis* may inhibit the POD activities, leading to slow anthocyanin degradation, while the high anthocyanin degradation POD activities in *B. calycina* may be related to the absence of these substances, leading to dramatic colour change.

In summary, to dissect the dramatic difference in anthocyanin degradation between *B. calycina* and *R. chinesis*, we found high levels of tannins, particularly the gallated catechins in the petals of *R. chinesis* but not in *B. calycina*. ECG and GCG showed high inhibition efficacy to PODs and may protect the pigments from enzymatic degradation. This finding may open a new approach to anthocyanin stabilization, and may enable improvements in crop pigmentation via induction of the accumulation of gallated catechins in plant cell vacuoles.

## 4. Materials and Methods

### 4.1. Plant Materials

*Brunfelsia calycina* flower petals at four stages (bud, dark purple, light purple and white, later indicated as stage 1, 2, 3 and 4 in Figure 1A) were collected in the campus of South China Agricultural University, Guangzhou City, south-east of China (23°7′ N, 113° E). *Rosa chinensis* flower petals at four stages (buds, partially open, fully open and initial senescent, later indicated as stage 1, 2, 3 and 4 in Figure 1A) were collected from the potting plants bought from Guangzhou Lingnan flower market and grown in the campus of South China Agricultural University. The sampled petals were either immediately subjected to analysis or frozen in liquid N_2_, and stored at −80 °C prior to measurement.

### 4.2. Measurement of Anthocyanin Content

One gram petals of the two plants at each indicated stages were sliced and immersed in 8 mL of 0.15 M HCl in water for anthocyanin extraction. The extraction was repeated until the solution was colourless and the red extracts were collected, combined and made to 20 mL using 0.15 M HCl as described by Zhang et al. [6]. Anthocyanin concentration of the extracts was determined by a pH-differential method [41]. The extract (1 mL) was diluted either in 4 mL of 0.4 M KCl–HCl buffer (pH 1.0) or in 4 mL of 0.4 M citric acid-Na_2_HPO_4_ buffer (pH 5.0) respectively. The absorbance of the dilutions was measured by a spectrophotometer (Shimadzu UV-2450, Kyoto, Japan) at 510 nm. Anthocyanin concentration was calculated as cyanidin-3-glucoside as described by Wrolstad et al. [41].

### 4.3. Measurement of Total Phenolic and Tannin Content

The extraction and determination of the contents of total phenolic compounds and tannins were carried out using Folin–Ciocalteu procedure [42] by using tannic acid (Sigma-Aldrich, Saint Louis, MO, USA) as standard according to Hagermann et al. [43], with minor modification. One gram of ground sample powder was homogenized in 40 mL of aqueous acetone (70%, *v*/*v*) and then subjected to an ultrasonic water bath for 20 min at room temperature, then the mixture was centrifuged for 10 min at 3500× *g* at 4 °C. The residue was re-extracted twice as above by 20 mL of the aqueous acetone. All supernatants were combined and made to 100 mL using the aqueous acetone. The total phenolic content was determined by mixing 0.05 mL of the extract with 0.45 mL distilled water and 0.25 mL of 1 M Folin–Ciocalteu reagent (Sigma-Aldrich) and 1.25 mL of 20% (*w*/*v*) sodium carbonate. The mixture was incubated at room temperature for 40 min, and the absorbance recorded at 725 nm. The total phenolic content in the petals was determined based on comparison to a standard curve generated using tannic acid from 0 to 20 μg/mL [42,43].

The remaining phenolic content was determined similarly after addition of 5% (*w*/*v*) of polyvinylpolypyrrolidone (PVPP) to remove the tannins from the extract. Tannin content was determined by subtracting the content of the remaining phenolic content from the total phenolic content [42,43].

### 4.4. Tissue Cell Sap pH Measurement and In Vivo Vacuolar Acidity Prediction by Neutral Red Staining and Microscopy

Cell sap pH (correlating to changes in vacuolar pH) in petals was determined by grinding the petals in liquid nitrogen and adding distilled water at a ratio of 0.1 g tissue to 1 mL H_2_O according to Vaknin et al. [8]. The pH of the homogenate was measured by a pH meter (Satorious, Göttingen, Germany). NR (neutral red, Sigma-Aldrich) was used for the acidity prediction of the vacuoles of the petal cells according to Poustka et al. [44]. The epidermis of the petals was layered in a drop of NR solution (20 mg/L) on a glass slide and incubated for 20 min at room temperature. The NR-stained and non-stained epidermal cells were observed by a light microscopy (Leica Microsystems, Wetzlar, Germany). All images were further processed using Adobe Photoshop software (Adobe Photoshop CS4, San Jose, CA, USA).

### 4.5. Vanillin Staining of Tannins in the Petals

Discs (2 mm × 2 mm) of *R. chinensis* and *B. calycina* petals were respectively fixed in 2% glutaraldehyde, 4% paraformaldehyde and 100 mM phosphate buffer (PBS; pH 7.2) for 12 h, washed three times for 30 min in 100 mM PBS, pH 7.2, and dehydrated in 30%, 50%, 70%, 80%, 85%, 90%, 95% and 100% (*v*/*v*) of ethanol gradient at 4 °C. The samples were infiltrated with LRWhite via three intermediate steps at 2:1, 1:1, and 1:2 (*v*/*v*) mixture of ethanol: LRWhite (12 h for each step). Finally, the mixture was replaced by pure LRWhite, kept for 12 h at −20 °C, and then changed with fresh LRWhite and kept for one day at −20 °C. Sections (2-mm thick) were incubated as described by Aastrup et al. [45] in a solution of 1% (*w*/*v*) vanillin and 6 N HCl at room temperature for 1 h. Observations and photographs were done on a light microscope (Optiphot, Nikon, Tokyo, Japan). Vanillin turns red upon binding to flavan-3,4-diols (leucoanthocyanidins) and flavan-4-ols (catechins), which are present either as monomers or as terminal subunits of condensed tannins (proanthocyanidins) [46].

### 4.6. Quantification of Tannin Constituents by HPLC

Tannins were extracted as described by Rzeppa et al. [47]. One gram of freeze-dried sample was ground into fine powder and homogenized in 15 mL *n*-hexane by vortexing for 30 s and shaking for 10 min. After centrifugation at 10 °C, 8000× *g* for 10 min, all the remaining organic solvent was removed under a nitrogen stream. Tannins were extracted with 15 mL aqueous acetone (70%, *v*/*v*) containing benzothiadiazole (BTH; 1%, *w*/*v*) by sonication for 10 min and shaking for 15 min. After centrifugation, the supernatant was transferred into a 50-mL volumetric glass tube. The residue was re-extracted as above and the supernatants were combined and made to 50 mL using the aqueous acetone. Then, 100 μL were dried under a nitrogen stream and re-dissolved in 1 mL ethanol/water (60:40, *v*/*v*). The solutions were then filtered through 0.45-μm polyvinylidene difluoride (PVD) membranes (Anpel Scientific instruments, Shanghai, China), and transferred to a vial before injection in a HPLC system with a diode array detection at 280 nm (Agilent Technologies, Santa Clara, CA, USA). A total of 5 μL was loaded onto an Eclipse XDB (extra dense bonding)-C18 column (4.6 × 250 mm, 5 μm, Agilent Technologies). Elution was carried out with a linear gradient of 0.2% (*v*/*v*) formic acid (B) and methanol (A) over 25 min, from 19.5% A, 80.5% B to 44.0% A, 56.0% B at a flow rate of 1 mL/min, followed by washing and reconditioning of the column. The tannin constituent peaks were identified by comparison with standards of catechin and its derivates (Sigma-Aldrich), including C ((+)-catechin), EC ((–)-epicatechin), GCG ((–)-gallocatechin-3-gallate), ECG ((–)-epicatechin-3-gallate), EGCG ((–)-epigallocatechin-3-gallate), and CG ((–)-catechin-3-gallate). The contents of the tannin constituents in the extraction were calculated based on the comparison of the identified peak to standard curves.

### 4.7. Extraction of Crude Enzyme, SDS-PAGE and Immunodetection

Petal samples of 2 g were ground to fine powder with liquid N_2_ and crude enzyme was extracted by homogenizing the sample powder with 8 mL of 0.1 M potassium phosphate buffer (KPB; pH 7.0), containing 15% (*w*/*v*) PVPP and 80 μL of protease inhibitor (1 tablet of complete protease inhibitor (Roche, Mannheim, Germany) was dissolved in 10 mL KPB). The homogenate was centrifuged for 20 min at 12,000× *g* and 4 °C, and the supernatants were collected as crude enzyme extract or soluble fraction of the protein extract [48]. For the insoluble fraction extraction, the residue from the above extraction was resuspended and homogenized in the above KPB containing 8 M of urea. The homogenate was centrifuged for 20 min at 12,000× *g* and 4 °C, and the supernatants were collected as insoluble fraction of the protein extract.

The enzyme extract was denatured by 10 min of boiling in Laemmli’s sample buffer prior to separation using 10% (*w*/*v*) sodium dodecyl sulfate-polyacrylamide gel electrophoresis (SDS-PAGE) following standard conditions [49]. The gel was stained with Coomassie Brilliant Blue R-250 (Sigma-Aldrich) to check the quality and purity of the proteins. For the following activity assays in-gel, semi-native PAGE of the crude enzyme extracts or the purified enzyme were separated on a 10% SDS-PAGE without protein sample boiling. The gels were rinsed twice with respective reaction buffers for 5 min to remove SDS.

For immunodetection, 4 μg of the soluble fraction or the insoluble fraction proteins from *B. calycina* and *R. chinensis* at stages 3 and 4 were resolved by 10% SDS-PAGE and electroblotted onto a nitrocellulose membrane (GE Healthcare, Munich, Germany) following standard conditions [49]. The membranes were then incubated with the polyclonal anti-Horseradish POD antibody (Agisera Antibody, Vännäs, Sweden) and detected using a StarSignal Chemiluminescent Assay Kit (GenStar, Beijing, China) and visualized by ChemiDoc™ MP imaging system (BIO-RAD, Hercules, CA, USA).

### 4.8. Peroxidase Activity Assay

According to Zhang et al. [7], peroxidase (POD) activity was measured using guaiacol (Sigma-Aldrich) as substrate in a reaction mixture (3 mL) containing 0.05 mL crude enzyme extract, 2.75 mL 0.05 M phosphate buffer (pH 7.0), 0.1 mL of 5 mM H_2_O_2_ and 0.1 mL of 400 μM guaiacol. As described by Chakraborty et al. [50], the increase in absorbance due to oxidation of guaiacol in the reaction mixture was monitored at 470 nm (ε (molar extinction coefficient) = 6740 M^−1^·cm^−1^ (mol·L)^−1^·cm^−1^)) for 2 min by a spectrophotometer (Shimadzu 2450-UV, Kyoto, Japan). POD activity was expressed as μmol/min/g FW (1 μmol of substrate conversion/min/g FW).

In-gel POD activity assay was carried out by immersing the semi-native PAGE gel described above in 15 μM guaiacol substrate with 0.17 mM H_2_O_2_ in 0.05 M PBS (pH 7.0).

### 4.9. Polyphenol Oxidase Activity Assay

Based on the method by Jiang [51], with minor modification, PPO activity was measured using 4-methylcatechol (Sigma-Aldrich) as a substrate in a reaction mixture (3 mL) containing 0.2 mL crude enzyme extract, 2.7 mL 0.05 M phosphate buffer (pH 7.0), 0.1 mL of 10 mM 4-methylcatechol. The increase in absorbance due to oxidation of 4-methylcatechol in the reaction mixture was monitored at 410 nm (ε = 1300 M^−1^·cm^−1^, given by Eichlerová et al. [52]) for 2 min by a spectrophotometer (Shimadzu 2450-UV). PPO activity was expressed as μmol/min/g FW (1 μmol of substrate conversion/min/g FW).

Activity assay of PPO in gel was carried out with 4-methylcatechol after native-PAGE according to Pinto et al. [53]. After semi-native electrophoresis as described above, the PPO activity was detected by staining the gel with 50 mM sodium phosphate buffer (pH 7.0) containing 5 mM 4-methylcatechol until the bands were visualized.

### 4.10. Anthocyanin Degradation Enzyme (ADE) Substrate Preparation and Activity Assay

Anthocyanins in *B. calycina* and *R. chinensis* flower petals were respectively extracted in 0.3 M HCl aqueous solution and the extraction was filtered through Whatman No.1 paper. The filtrates were partially purified by Amberlite XAD-7 resin (Sigma-Aldrich, Saint Louis, MO, USA) column (1.5 cm × 40 cm) according to Zhang et al. [54]. The fractions containing anthocyanins were concentrated using a rotary evaporator (Heidolph, Schwabach, Germany). The content of the concentrated anthocyanins was determined using the pH-differential method as described above. Substrates of the two flowers for ADE activity assay were prepared respectively by diluting the concentrated anthocyanin extract to a concentration of about 0.05 M in 0.2 M sodium acetate buffer (pH 4.0).

ADE activity assay was carried out according to Fang et al. [17]. Crude enzyme extracts (0.1 mL) of the two flowers were added to the respective 2-mL ADE substrates. The mixture was incubated for 20 min at 40 °C. The reaction was terminated by adding 2 mL 0.1 M HCl in methanol. The rates of anthocyanin degradation were determined by the decrease in absorbance at 530 nm when compared to the reaction set up in parallel with denatured enzyme that had been boiled for 10 min. Enzyme activity was expressed as the degradation of 1 μmol of cyanidin-3-glucoside (ε = 29,600 M^−1^·cm^−1^) per hour at 40 °C. ADE activity was expressed in μmol/h/g FW.

Activity gel staining of ADE using the anthocyanin substrates with or without H_2_O_2_ was after native-PAGE respectively. After semi-native electrophoresis as described above, the gel was rinsed twice with 0.2 M sodium acetate buffer (pH 4.0) for 5 min to remove SDS, and immersed in the same buffer containing 0.05 M partially purified anthocyanin substrates with 2 mM H_2_O_2_ or without H_2_O_2_ until the bands were visualized.

### 4.11. POD Purification from B. calycina Flower Petals

Eighty grams of frozen *B. calycina* flower petals were ground and immediately homogenized in 300 mL of 0.1 M KPB (pH 7.0) containing 8.6 mM dithiothreitol (DTT), 5 mM ethylenediaminetetraacetic acid (EDTA), 1 mM phenylmethylsulfonyl fluoride (PMSF) and 5% (*w*/*v*) PVPP, followed by centrifugation at 13,000× *g* for 20 min. The extract was fractionated further by precipitation in 20% to 70% saturation of ammonium sulfate and centrifugation at 13,000× *g* for 20 min. The precipitate was re-suspended in 0.1 M KPB (pH 7.0) containing 8.6 mM DTT, 1 mM EDTA, 0.2 mM PMSF and dialyzed overnight against 0.01 M KPB (pH 7.0) containing 2.2 mM DTT. The dialyzed solution was applied to a DEAE-Sepharose (GE Healthcare Bio-Sciences AB, Uppsala, Sweden) column (1.5 cm × 50 cm) previously equilibrated with 0.01 M KPB (pH 7.0) containing 1.1 mM DTT. POD was eluted from the column at a flow rate of 0.5 mL/min. Fractions of 2 mL were collected and assayed both for POD activity and protein concentration (A280). The flow-through fractions that contained high enzyme activity were combined and concentrated by Amicon Ultra-15 Centrifugal Filter Units (Merck KGaA, Darmstadt, Germany). The concentrated enzyme solution of 4 mL was loaded onto a Sephadex G-200 (Sigma-Aldrich, Saint Louis, MO, USA)) column (1.5 cm × 70 cm) previously equilibrated with 0.01 M KPB (pH 7.0), and eluted with the same buffer at a flow rate of 0.33 mL/min. Fractions of 1 mL were collected and assayed for POD activity and A280, and the fractions with the highest activity and the highest ratio of activity to protein were pooled.

### 4.12. Measurement of IC50 and Km Values of Tannin Constituents on B. calycina POD Activity

The half maximal inhibitory concentration (IC50) of the tannin constituents on the purified *B. calycina* POD was tested by adding the tannin constituents to the POD activity assay reaction mix as described above. Then, 0, 0.5, 5, 10, 15, 25 and 50 μg/mL of C, EC, GCG, and ECG were respectively added to the reaction mixtures with 400 μM guaiacol, 5 mM H_2_O_2_ and 0.615 μg *B. calycina* POD. The POD activities were measured as described above and the inhibition ratio was obtained by calculating the percentage of the decrease in the activity due to the addition of the tannin constituents, to the activity without addition.

The Km values of the purified *B. calycina* POD to guaiacol, C and EC were determined by extrapolation of Lineweaver and Burk plots. Since this was the first time for C and EC to be tested as substrates of POD, the spectra of C and EC before and after oxidation by POD in the presence of H_2_O_2_ were scanned by spectrophotometer (Shimadu UV-2450, Kyoto, Japan) from 200 to 600 nm. Absorbance peaks due to oxidation of C and EC by POD were recorded at 430 nm. The absorbance increase of 0.01 at 430 nm per minute was designated as 1 activity unit. Concentrations of 50, 100, 150, 200, 250, 300, and 400 μM of the three substrates were used to detect the POD activities (v) as described above. The Km values were obtained by the Lineweaver and Burk plots of the activities (v) and the concentrations of the substrates (s).

### 4.13. Determination of the Effect of Tannin Constituents on Colour Intensities of Anthocyanins

Anthocyanin extracts (0.05 mM) of *B. calycina* and *R. chinensis* flowers were prepared in 0.4 M Citric acid-disodium hydrogen phosphate buffer at pH 3.0. Then, separate additions of 0, 100, and 500 μg/mL of C, EC, ECG and GCG were added to the anthocyanin solutions respectively. The absorbance at 510 nm of each mixture was recorded to determine the effect of tannin constituents on the colour intensity of the anthocyanin extracts.

### 4.14. Statistical Analyses

The contents and activities were measured with three biological replicate samples. Data were presented as means ± standard error of mean (SEM). Means were compared by unpaired *t* test (*p* < 0.05). The analysis was performed with the GraphPad QuickCalcs online software (Prism 7, GraphPad Software, San Diego, CA, USA).

## Supplementary Materials

Supplementary materials can be found at http://www.mdpi.com/1422-0067/18/4/699/s1.
