# Supplementary material for: Characterization of Active Anthocyanin Degradation in the Petals of Rosa chinensis and Brunfelsia calycina Reveals the Effect of Gallated Catechins on Pigment Maintenance
Source: Int J Mol Sci. 2017 Mar 25;18(4):699. doi: 10.3390/ijms18040699 (PMC5412285; doi:10.3390/ijms18040699)
Supplement: Supplementary file 1 [file ijms-18-00699-s001.pdf]

Characterization of Active Anthocyanin Degradation in the Petals of *Rosa chinensis* and *Brunfelsia calycina* Reveals the Effect of Gallated Catechins on Pigment Maintenance

Honghui Luo, Shuangfan Deng, Wei Fu, Xin Zhang, Xuelian Zhang, Zhaoqi Zhang and Xuequn Pang

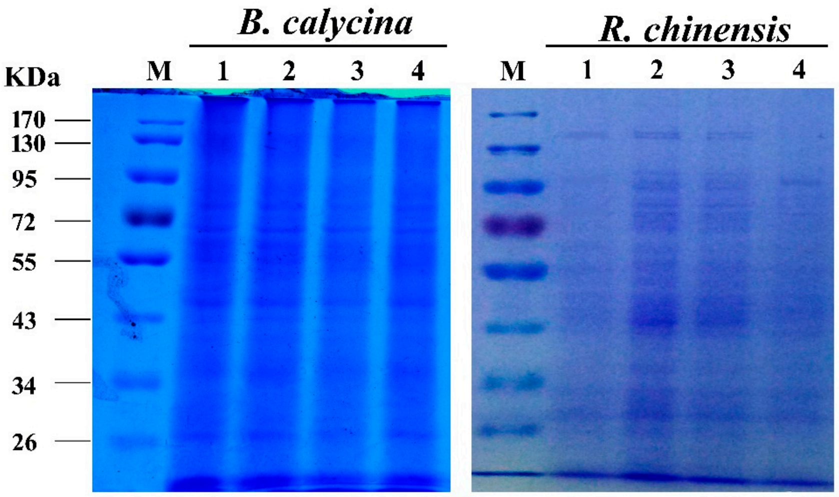

Figure S1. SDS-PAGE of the crude enzyme extracts of *Brunfelsia calycina* and *Rosa chinensis* flowers for the in-gel activity assay. As shown in Figures 2 and 3. Lane M indicates the protein markers, while lanes 1-4 show four samples collected at flower developmental stages 1, 2, 3, 4.

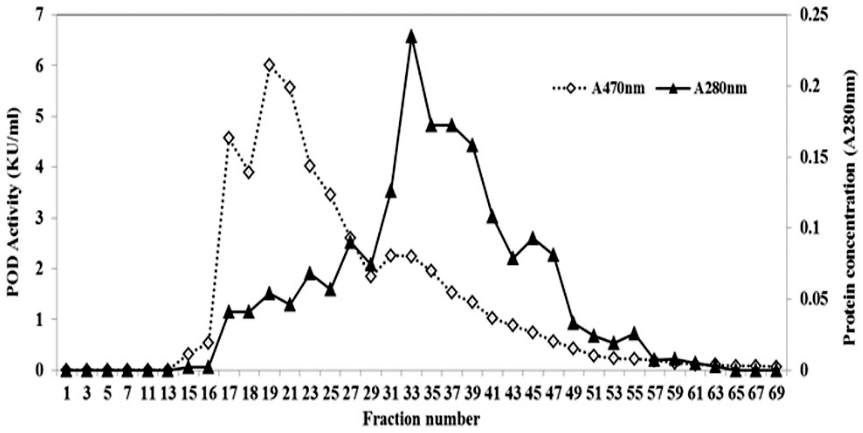

Figure S2. Elution profile of the purification of *Brunfelsia calycina* peroxidase (POD) by Sephadex G-200 chromatography.
